# Supercritical Fluid Extraction of Lutein from *Scenedesmus almeriensis*

**DOI:** 10.3390/molecules24071324

**Published:** 2019-04-04

**Authors:** Sanjeet Mehariya, Angela Iovine, Giuseppe Di Sanzo, Vincenzo Larocca, Maria Martino, Gian Paolo Leone, Patrizia Casella, Despina Karatza, Tiziana Marino, Dino Musmarra, Antonio Molino

**Affiliations:** 1 ENEA, Italian National Agency for New Technologies, Energy and sustainable economic Development, Department of Sustainability—CR Portici, P. Enrico Fermi, 1, 80055 Portici (NA), Italy; smehariya@gmail.com (S.M.); angela.iovine@unicampania.it (A.I.); patrizia.casella@enea.it (P.C.); 2 Department of Engineering, University of Campania “L.Vanvitelli”, Real Casa dell’Annunziata, Via Roma 29, 81031 Aversa (CE), Italy; karatza@irc.cnr.it (D.K.); tiziana.marino@yahoo.it (T.M.); dino.musmarra@unicampania.it (D.M.); 3 ENEA, Italian National Agency for New Technologies, Energy and sustainable economic Development, Department of Sustainability—CR Trisaia, SS Jonica 106, km 419+500, 7026 Rotondella (MT), Italy; giuseppe.disanzo@enea.it (G.D.S.); vincenzo.larocca@enea.it (V.L.); maria.martino@enea.it (M.M.); 4 ENEA, Italian National Agency for New Technologies, Energy and sustainable economic Development, Department of Sustainability—CR Casaccia, Via Anguillarese 301, 00123 Rome (RM), Italy; gianpaolo.leone@enea.it

**Keywords:** microalgae, lutein, carotenoids, fatty acids, food additives, dietary supplements, pretreatment, recovery, purity

## Abstract

Lutein has several benefits for human health, playing an important role in the prevention of age-related macular degeneration (AMD), cataracts, amelioration of the first stages of atherosclerosis, and some types of cancer. In this work, the *Scenedesmus almeriensis* microalga was used as a natural source for the supercritical fluid (SF) extraction of lutein. For this purpose, the optimization of the main parameters affecting the extraction, such as biomass pre-treatment, temperature, pressure, and carbon dioxide (CO_2_) flow rate, was performed. In the first stage, the effect of mechanical pre-treatment (diatomaceous earth (DE) and biomass mixing in the range 0.25–1 DE/biomass; grinding speed varying between 0 and 600 rpm, and pre-treatment time changing from 2.5 to 10 min), was evaluated on lutein extraction efficiency. In the second stage, the influence of SF-CO_2_ extraction parameters such as pressure (25–55 MPa), temperature (50 and 65 °C), and CO_2_ flow rate (7.24 and 14.48 g/min) on lutein recovery and purity was investigated. The results demonstrated that by increasing temperature, pressure, and CO_2_ flow rate lutein recovery and purity were improved. The maximum lutein recovery (~98%) with purity of ~34% was achieved operating at 65 °C and 55 MPa with a CO_2_ flow rate of 14.48 g/min. Therefore, optimum conditions could be useful in food industries for lutein supplementation in food products.

## 1. Introduction

Lutein (C_40_H_56_O_2_) belongs to the xanthophylls group, composed of carotenoids characterized by oxygen atoms or hydroxyl groups in their chemical structure. Similarly to the other carotenoids, lutein is located in the chloroplast, where it performs the capture of selective wave lights and the transfer of energy to chlorophyll. The principal end effect is to protect the cell from the formation of harmful reactive species. Thanks to its action against photodegradation and its ability as an antioxidant and coloring agent, lutein can be used as a food additive, a supplement in the nutraceutical field, and as a conditioning compound in cosmetics. Lutein is mainly used to confer color to poultry and egg yolks. In Europe, it has been classified and authorized as a food additive, from Regulation (EU) No 231/2012 with E161b classification [1]. In addition, it is marketed as a dietary supplement for its beneficial effects on the visual apparatus [2]. Along with other carotenoids, such as zeaxanthin, lutein is contained in the central zone of the retinal eye named macula and its density decrease can cause macular degeneration of the eye [3]. The prevention of this disease could be avoided by the assimilation of a daily lutein dose of 0–2 mg/kg body weight, as suggested by World Health Organization (WHO) [4,5].

The use of lutein as a food additive as well as a dietary supplement represents about 90% of its worldwide market [4,6]. In the last few years, this market has shown a growing trend, from US$105.1 million in 2006 to $233 million in 2010, with a forecast of $308 million in 2018 [6]. Lutein, as used in the international market, is extracted from the petals of *Tagetes erecta* flower by using hexane as an extractive solvent. The produced oleoresins are marketed or refined by a saponification process to convert esters into free forms of lutein [7]. However, the production of lutein from *T. erecta* has some drawbacks connected to the seasonality of flowering, land use, and high water consumption, i.e., about 50–80 m^3^ for each kg of lutein produced [8].

Alternative natural producers of lutein are microalgae, autotrophic aquatic microorganisms able to produce carotenoids [9]. Microalgae cultivation usually occurs in large tanks (open ponds) and photo bioreactors [10]. This last technology allows us to overcome the issues related to the competition for land that could be used for human nutrition. In addition, microalgae for lutein production is advantageous due to the higher lutein content than *T. erecta* flowers, leading to beneficial effects on productivity—up to 5-fold greater than *T. erecta* [8].

Some microalgae species, such as Muriellopsis sp., *Scenedesmus almeriensis*, *Chlorella protothecoides*, *C. zofingiensis*, *Botryococcus braunii*, *Neospongiococcus gelatinosum*, and *Chlorococcum citriforme* were studied because they are capable of accumulating lutein [11]. Among them, *S. almeriensis* is one of the most suitable since it can accumulate lutein in the range of 0.2–0.5% on dry weight [12,13]. Currently, *S. almeriensis* is not cultivated at an industrial scale due to some critical issues associated with the higher cost of production [14]. Furthermore, by using photo-bioreactors, the oxygen feeding can cause its saturation in the culture medium as well as the walls reactor fouling [12,15,16,17]. This phenomenon is related to the production of ascorbic acid and exopolysaccharides (EPS), which contain mono-saccharides such as glucose, fructose, xylose, and mannose [18]. In order to reduce this, it is important to design a good aeration and agitation system within the photo bioreactor, optimizing the retention times of the microalga in the reaction device.

The literature proposes different lutein extraction technologies from *S. almeriensis* but the findings are not sufficient to establish a clear methodological and a defined approach for the evaluation of extraction costs, yield, and quality of the final extracts [19,20,21,22,23,24,25,26].

Therefore, the aim of this study was to evaluate the effect of extraction temperature, pressure, and CO_2_ flow rate on the recovery and purity of lutein using supercritical fluid extraction.

## 2. Results and Discussion

### 2.1. Effect of Matrix Solid-Phase Dispersion

**Figure 1:**
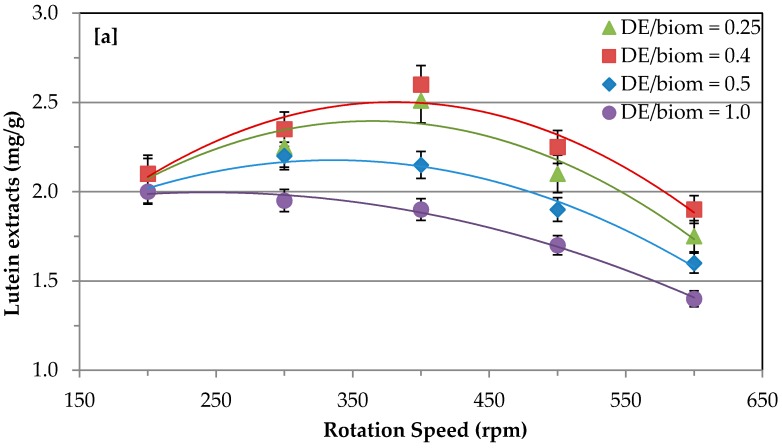

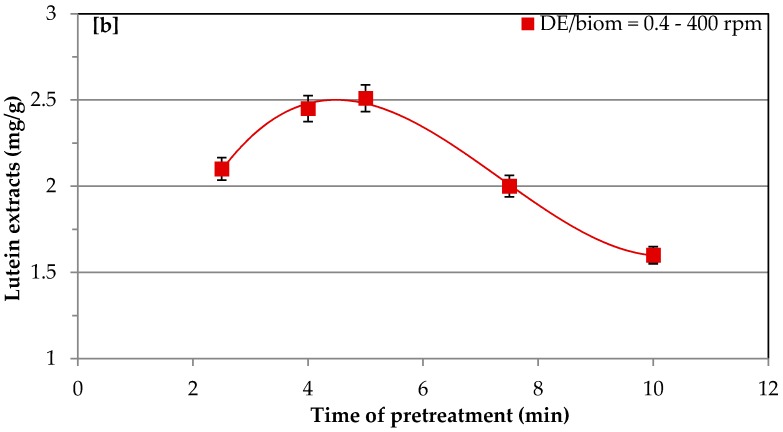
Role of different pretreatment conditions on extraction efficiency of lutein. (**a**) Effect of DE/biomass mixing ratio and rotation speed; (**b**) effect of pretreatment time at 400 rpm with a 0.4 DE/biomass ratio.

Matrix solid-phase dispersion (MSPD) of microalgae biomass was carried out using a ball miller to enhance the extraction efficiency of intracellular compounds [19,24,27]. Therefore, the pretreatment conditions were optimized to enhance the recovery of lutein from *S. almeriensis*. The mixing ratio of diatomaceous earth (DE) and biomass played a significant role during the pre-treatment. The DE provides better support during the MSPD, due to the wide pore size and low surface activity as compared to alumina; therefore, it was selected as the inert material during MSPD. The appropriate mixing of DE and biomass provided the optimum bulk density, and, consequently, the mechanical force to break down the rigid cell wall. Among the varied mixing ratios of DE/biomass (0.25–1 DE/biomass) at different rotation speeds, 0.4 DE/biomass resulted in the highest lutein extraction. The pre-treatment degree at the optimum rotation speed generated an efficient mechanical force, which implied cell wall disruption and reduction of biomass. Among the different degrees of pre-treatment, cell disruption at 400 rpm led to lutein maximum extraction yield corresponding to 2.6 mg/g, which was around 85.6% of the theoretical content. The optimum stirring rate provides the effective mechanical force, which leads to homogenously break the microalgae cell. Therefore, the degree of MSPD needs to be optimized for each microalga due to their varied chemical composition of intracellular molecules and the cell wall structure of microalgae biomass [24,26]. In the second step, the pre-treatment time (2.5–10 min) was optimized to enhance the lutein recovery at 400 rpm with 0.4 DE/biomass ratios. Increasing the pre-treatment time from 2.5 to 5 min gradually increased the pre-treatment efficiency, followed by lutein recovery, and thereafter led to a decrease in the efficiency. However, a longer MSPD time could break the lutein molecules; therefore, the recovery of lutein decreased. Figure 1a,b shows that the pre-treatment of *S. almeriensis* biomass was necessary to enhance the lutein extraction. Cerón et al. [19] tested different pre-treatment methods to enhance the lutein recovery from *S. almeriensis* biomass and found that bead mill pre-treatment for 5 min was the optimal condition with regard to industrial applications. It was also reported that during the bead miller pre-treatment the appropriate mixing of biomass and alumina (1:1 *w*/*w*) was important to increase the lutein extraction [19]. In addition, several reports suggest that pretreatment of microalgae biomass at optimum condition is essential to enhance the extraction of intracellular compounds [28,29,30,31]. In this work, the effective pretreatment of *S. almeriensis* biomass was achieved at 400 rpm for 5 min with 0.4 DE/biomass ratios to assist with the extraction of intracellular molecules.

### 2.2. Effect of SF-CO_2_ Extraction Parameters on Extract Yield

In the first instance, the influence of the SF-CO_2_ parameters (i.e., temperature, pressure, and CO_2_ flow rate) on the extraction yield was investigated. Table 1 summarizes the cumulative quantitative extracts yield and total lipids yield after the complete extraction (at 120 min). At 50 °C, the increase in pressure from 25 to 55 MPa showed the positive influence on extraction yield as well as on total lipids yield at CO_2_ flow rates of 7.24 and 14.48 g/min. A similar trend was observed at 65 °C, excluding extraction yield at 40 MPa with a CO_2_ flow rate of 7.24 g/min.

Increasing the pressure at constant temperate enhanced the diffusivity of the solvent, which allowed us to improve the solubilization of intracellular compounds. By operating at 65 °C, with a CO_2_ flow rate of 14.48 g/min and a pressure of 40 MPa, the highest total lipids yield was registered, while the highest extraction yield was obtained under the same operational conditions of temperature and pressure, but with a lower CO_2_ flow rate (7.24 g/min). In this case the extraction yield was around 1.7-fold lower at 65 °C, and 40 MPa at a CO_2_ flow rate of 14.48 g/min, while the total lipids yield was 1.4-fold higher as compared to the CO_2_ flow rate of 7.24 g/min. These results indicated that less contact time between the solvent and the solute enhanced the selectivity towards total lipids, whereas the extraction yield was lower. An analysis of Table 1 indicates that increasing the temperature from 50 to 65 °C has a positive influence on the lipid extraction at each tested pressure and CO_2_ flow rate. However, a higher temperature enhanced the extraction yield with a CO_2_ flow rate of 7.28 g/min, while caused a decrease when operating at 14.48 g/min CO_2_. These results indicated that 65 °C has higher selectivity towards lipids at a CO_2_ flow rate of 14.48 g/min with higher purity because the extraction yield was lower. Therefore, 65 °C and a CO_2_ flow rate of 14.48 g/min should represent better choices to attain higher extractability of lipids with higher purity.

### 2.3. Effect of SF-CO_2_ Extraction Parameters on Lutein Recovery and Purity

**Figure 2:**
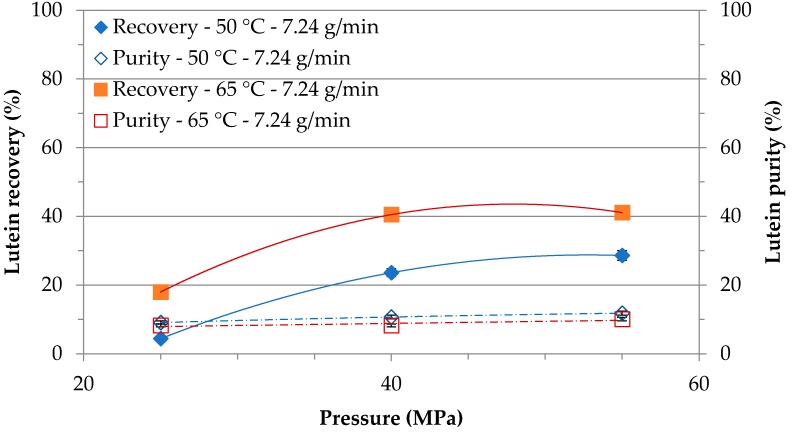
Influence of SF-CO_2_ extraction temperature and pressure at CO_2_ flow rate of 7.24 g/min on the recovery and purity of lutein.

The influence of the extraction conditions on the lutein recovery and purity was analyzed in more detail. Figure 2 shows the cumulative lutein recovery and purity behavior in function of extraction pressure at 50 and 65 °C with a CO_2_ flow rate of 7.24 g/min after 120 min. The results highlighted that increasing the pressure from 25 MPa to 40 MPa led to a drastic improvement in lutein recovery—5.4- and 2.3-fold higher compared to at a lower pressure, at 50 and 65 °C, respectively. A further increase of pressure up to 55 MPa led to a slight increase in lutein recovery. This behavior was expected since the solubility of the intracellular compounds increases with pressure at a constant temperature due to the increase in the density of the solvent. From Figure 2 it can also be observed that increasing the temperature from 50 to 65 °C had a positive influence on lutein selectivity. At 25 MPa, the lutein recovery increased by 4.1-fold; at 40 and 55 MPa the lutein recovery improvement was 1.7- and 1.4-fold, respectively at 65 °C as compared to 50 °C. At a constant pressure, increasing the temperature enhanced lutein recovery due to the enhancement in the solubility of the intracellular compounds in the solvent, which caused the increase in the vapor pressure of lutein [22]. The optimum detected pressure and temperature improved the solvation power of the fluid due to their actions on the solvent density, which increased the extraction efficiency. On the other hand, pressure and temperature had no significant influence on the lutein purity at a CO_2_ flow rate of 7.24 g/min. The maximum lutein purity of 11.8% was achieved at 50 °C and 55 MPa, while a maximum lutein recovery of 41.1% was observed at 65 °C and 55 MPa with a CO_2_ flow rate of 7.24 g/min. In an earlier study, comparable lutein recovery was attained from *Haematococcus pluvialis* at 65 °C and 55 MPa with a CO_2_ flow rate of 3.62 g/min, while the maximum lutein recovery was achieved at 50 °C and 55 MPa with a CO_2_ flow rate of 3.62 g/min [32]. However, the biomass properties and the type of extractable target compounds were different in the studies, which may be correlated with the variation in the optimum SF-CO_2_ extraction conditions.

**Figure 3:**
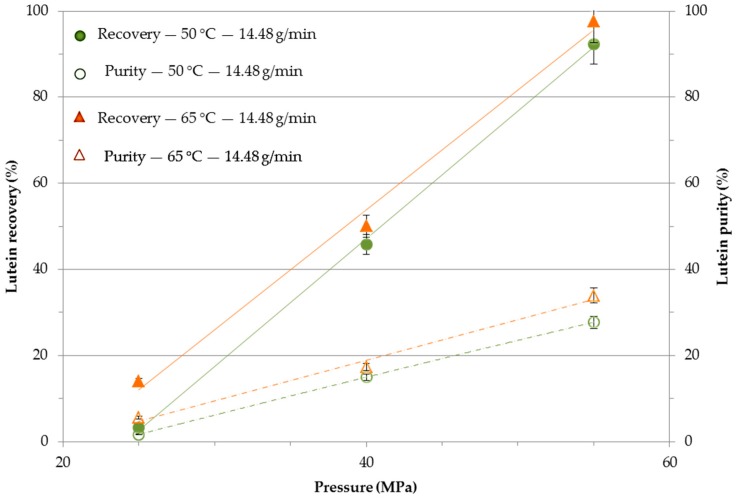
Influence of SF-CO_2_ extraction temperature and pressure at CO_2_ flow rate of 14.48 g/min on the recovery and purity of lutein.

Figure 3 reports the effect of extraction temperature and pressure on cumulative lutein recovery and purity at a higher CO_2_ flow rate, i.e., 14.48 g/min after 120 min. It can be seen from Figure 3 that the maximum lutein recovery and purity were obtained by fixing the pressure at 55 MPa and the temperature at 65 °C. This can be attributed to the increase of CO_2_ density, which enhanced its solvating power, thus favoring the solubilization of the lutein. An analysis of Figure 3 indicates that, at 20, 40, and 55 MPa, with a CO_2_ flow rate of 14.48 g/min, an increase in the temperature up to 65 °C slightly increased the lutein recovery and purity. However, a similar trend was observed at a CO_2_ flow rate of 7.24 g/min, although in this case the recovery and purity increase was high with respect to the pressure increase. Therefore, it could suggest that pressure had a strong effect on the selectivity of lutein, even though the CO_2_ flow rate significantly influenced the lutein recovery and purity. A CO_2_ flow rate of 14.48 g/min led to the maximum lutein recovery of 97.6% and purity of 33.9% at 65 °C and 55 MPa, which was 2.4- and 3.4-fold higher with respect to lutein recovery and purity at a CO_2_ flow rate of 7.24 g/min at the same extraction temperature and pressure. The increase in the recovery and purity was caused by the lower solute-solvent contact time, which may increase the selectivity of lutein during SF-CO_2_ extraction. Similar results were obtained by Macías-Sánchez et al. [22], who reported that it was necessary to work at a pressure of 400 bar and a temperature of 60 °C to obtain a significant yield in the extraction of intracellular compounds. Yen et al. [33] observed that the maximum lutein recovery of 76.7% could be obtained from *Scenedesmus* biomass using SF-CO_2_ extraction at 400 bar, 70 °C with ethanol as the co-solvent. However, the co-solvent helped to modify both the polarity of CO_2_ during SF-CO_2_ extraction and the extraction efficiency, but negatively affected the purity of the target extractable intracellular compounds [25]. However, in this study 97.6% lutein recovery was obtained with a purity of 33.9% at 65 °C and 55 MPa with a CO_2_ flow rate of 14.48 g/min without a co-solvent.

### 2.4. Effect of SF-CO_2_ Parameters on FAs Yield

**Figure 4:**
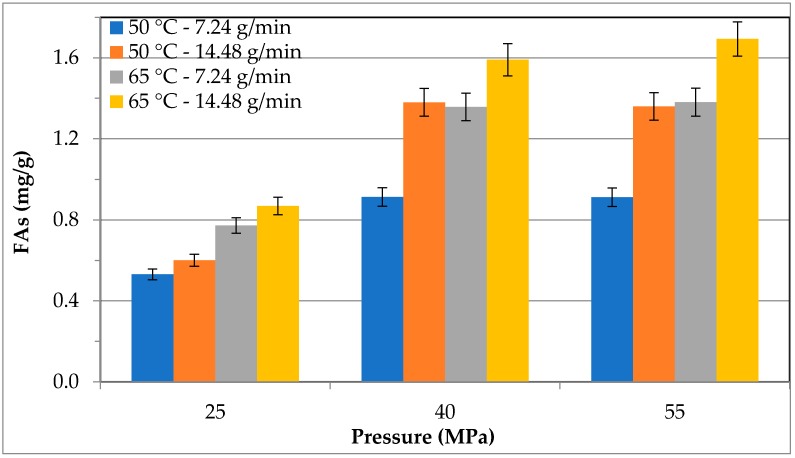
Influence of SF-CO_2_ extraction on fatty acid extraction yield.

Figure 4 shows the cumulative extraction yields of FAs obtained during the SF-CO_2_ extraction from the *S. almeriensis* biomass at different operating pressures (25, 40, and 55 MPa) and temperatures (50, 65 °C) with a CO_2_ flow rate of 7.24 or 14.48 g/min. All the results corresponded to an extraction time of 120 min. The extraction of FAs with SF-CO_2_ was increased with increasing temperature, pressure, and CO_2_ flow rate at a constant temperature/pressure/CO_2_ flow rate. At 50 °C, the maximum FAs yield was obtained at 40 MPa, which was 1.7-fold higher with respect to 25 MPa, while it slightly decreased at 55 MPa when a CO_2_ flow rate of 7.24 g/min was registered. However, the higher CO_2_ flow rate significantly improved the FAs yield at each fixed temperature and pressure. Therefore, the maximum effect of CO_2_ flow rate was observed at 50 °C and 40 MPa, which showed a 1.5-fold higher FAs yield at a CO_2_ flow rate of 14.48 g/min than at a CO_2_ flow rate of 7.24 g/min. A negligible effect of CO_2_ flow rate was found at 25 MPa; its influence at 55 MPa was lower if compared at 40 MPa and 50/65 °C. More precisely, the obtained FAs yield was the highest when working at 65 °C and 55 MPa. The extraction efficiency of FAs was gradually enhanced by increasing the pressure at 65 °C, which expanded the solvating power and led to a boost in the FAs extraction yield. Furthermore, by keeping the pressure and CO_2_ flow rate constant, the increase in extraction temperature enhanced the vapor pressure, which improved the extraction efficiency and enhanced the FAs yield [34,35]. Di Sanzo et al. [32], reported that at optimum temperature (i.e., 65 °C) the solvating power increased and the maximum FAs recovery could be obtained from *H. pluvialis* during SF-CO_2_ extraction at 65 °C and 55 MPa with a CO_2_ flow rate of 14.48. Crampon et al. [36] investigated the effect of SF-CO_2_ on oil extraction yield from *Spirulina platensis* and the results revealed a complex influence of temperature on solubility, which increased when temperature increased.

### 2.5. Effect of SF-CO_2_ Parameters on Extraction of Lipid Composition

The operating conditions (i.e., temperature, pressure, CO_2_ flow rate) significantly influenced the extraction efficiency of fatty acids, as reported in Table 2. The results showed that each SF-CO_2_ operating condition was more favorable for the extraction of arachidic acid (C20:0) and myristoleic acid (C14:1; ω-5) rather than other fatty acids. However, the maximum recovery of C14:1 (ω-5) (100%), palmitic acid (C16:0) (21%), stearic acid (C18:0) (16%), and γ-linolenic acid (C18:3; ω-6) (5%) was attained at 65 °C and 55 MPa with a CO_2_ flow rate of 14.48 g/min. Moreover, the same temperature and CO_2_ flow rate were promising for obtaining a higher recovery of arachidic acid (C20:0) (49%), cis-10-heptadecenoic acid (C17:0) (22%), and linoleic acid C18:2; ω-6) (38%) at 40 MPa. An analysis of Table 2 shows that the high temperature, pressure, and CO_2_ flow rate during SF-CO_2_ extraction have a negative influence on the extraction of palmitoleic acid (C16:1) and cis-9-octadecenoic acid (C18:1), while the extraction yield was higher at a lower operating temperature, pressure, and CO_2_ flow rate. The maximum recovery of C16:1 (8%) and C18:1 (11%) was achieved at 50 °C and 25 MPa with a CO_2_ flow rate of 7.24 g/min.

### 2.6. Comparison of Lutein, FAs, and Lipid Global Recovery at Different Operating Conditions

The effect of SF-CO_2_ extraction conditions was evaluated on lutein, lipids, and FAs from pre-treated dried biomass of *S. almeriensis* (Table 3). The maximum recovery of lutein, 2.97 mg/g (~98%), and FAs (15%) was achieved at 65 °C and 55 MPa with a CO_2_ flow rate of 14.48 g/min, while maximum lipid recovery (18%) was attained at 40 MPa. For cumulative lutein recovery, by increasing both pressure and temperature the extraction efficiency at CO_2_ flow rates of 7.24 and 14.48 g/min was improved, and similar observations were reported in earlier studies [25,32]. At a CO_2_ flow rate of 7.24 g/min, the increase of extraction pressure led to an increase in lipid recovery, while at a CO_2_ flow rate of 14.48 g/min, increased extraction pressures up to 40 MPa enhanced lipid recovery and future increased pressure caused a slight decline in lipid recovery. Furthermore, maximum lipid recovery was achieved at 65 °C and 40 MPa; a similar observation was reported in the literature [27].

### 2.7. Comparison of SF-CO_2_ with Other Methods of Lutein Recovery

Chen et al. [37] performed solvent extraction at 25 °C and 850 mbar with an extraction time of 20 min using acetone, ethanol, ether, hexane, and tetrahydrofuran (THF) for lutein recovery. The results showed that lutein recovery was 87.0% with THF > 77.1% with ether > 67.9% with ethanol > 64.7% acetone > 4.6% with hexane solvent. However, solvent extraction led to higher recovery, while reducing the purity of the extracted compound. However, Chen et al. [37] achieved maximum recovery of 87% using THF solvent, while in this study SF-CO_2_ showed 98% lutein recovery with a purity of 34%. The lutein extraction performance using different methods is compared with the literature reports in Table 4.

## 3. Materials and Methods

### 3.1. Microalgal Biomass

Experiments were carried out by using lyophilized *S. almeriensis* biomass collected from AlgaRes/University of Almeria, Spain, with a mesh particle size of 5–45 μm. The biomass was stored at −20 °C in a vacuum-sealed plastic bag to avoid changes in the chemical properties. The microalgae biomass was brought to normal atmospheric conditions before use for experimental activities. The chemical composition of lyophilized *S. almeriensis* are reported in Table 5, in terms of humidity, ash, total dietary fiber (TDF), carbohydrates, proteins, total lipids, FAs, and specifically lutein. The chemical characterization of biomass was carried out before extraction using standard methods [39].

### 3.2. Chemicals

The CO_2_ (99.999% purity) used for supercritical fluid extraction was provided by Rivoira, Milan, Italy. The standards (FAs) used for the GC calibrations were analytical grade and purchased from Sigma Aldrich, St. Louis, MO, USA. All the solvents used were of HPLC grade and purchased from Sigma Aldrich, USA.

### 3.3. Matrix Solid-Phase Dispersion of Biomass

In order to improve the extraction efficiency of intracellular compounds, the MSPD (mechanical pre-treatment) conditions were optimized in terms of biomass and DE mixing ratio, rotation speed, and pre-treatment time using a Retsch (Retsch Technology GmbH, Haan, Germany) PM200 planetary ball mill [26]. The jars of the mill were filled with ~2 g of *S. almeriensis* biomass and DE was added in a mixing ratio of 0.25, 0.4, 0.5, and 1.0 DE/biomass. In the first step of the experiment, pre-treatment was performed for 5 min at different rpm (200, 300, 400, 500, and 600 rpm). In the second step of the experiments the optimum ratio of DE/biomass and rpm was used to achieve the ideal pre-treatment time from 2.5, 4.0, 5.0, 7.5, and 10 min. The extraction efficiency of intracellular compounds, i.e., lutein and lipids, from mechanical pre-treatment of biomass was assessed using an accelerated solvent extractor (ASE) with hexane as the solvent at 50 °C and 100 bar. Each extraction was carried out using two extraction cycles, each of 10 min, for a total extraction time of 20 min; furthermore, the compounds was analyzed using u-HPLC and GC-FID, as discussed in subsequent sections.

### 3.4. CO_2_ Supercritical Extraction Experiments

**Figure 5:**
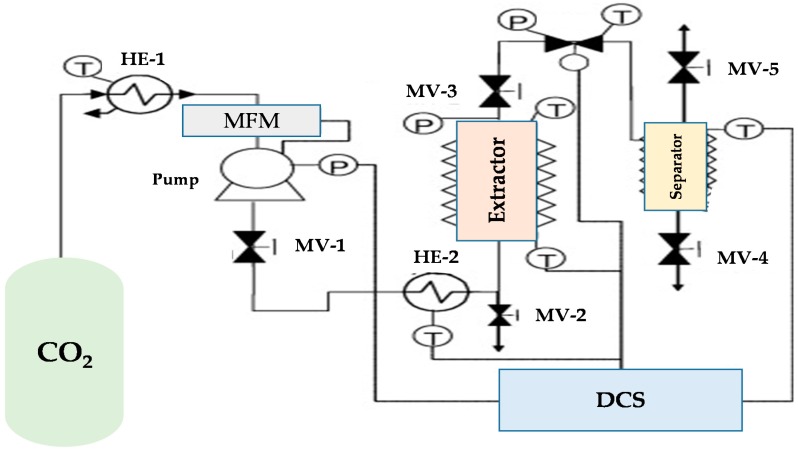
Diagram showing the flow in the extraction process with supercritical carbon dioxide. HE, heat exchanger; MFM: mass flow meter; MV: manual valve; DCS: distributed control system.

The SF-CO_2_ extraction apparatus used in the study was described in detail in our previous study [32] and the schematic is shown in Figure 5. The extraction unit had a heating capacity up to 250 °C and CO_2_ compression capacity of up to 68 MPa. The extraction unit can control the inlet and outlet pressure with an accuracy of 0.6 mbar, and the CO_2_ flow rate was controlled using a flow meter LPN/S80 ALG 2.5. The inlet flow rate was adjustable until 45 g/min and controlled using the expanded gas. The temperature was monitored using thermocouples, where inlet and outlet flow streams are controlled by micrometric valves. The biomass loading cylindrical vessel had a capacity of 50 mL (diameter = 1.35 cm, height = 35 cm), which was filled with ~2.8 g of pre-treated biomass (biomass: ~2 g), including DE (DE: ~0.8 g), and 44 g of glass beads with a size of 3 mm to increase the porosity and mass transfer of CO_2_ with microalgae and at the same time avoid biomass caking. The microalgae biomass had a density of 770 g/L, while the mixture of biomass and DE had a density of 570 g/L (mass of pre-treated biomass/volume). The bulk density of the pre-treated biomass and glass beads was maintained at 69 g/L for each extractive condition.

Furthermore, at the bottom of the extraction vessel, metal frit filters with a pore diameter of 5 μm were used. The extraction unit was equipped with acoustic and visual high-pressure alerts and, Sacofgas, Milan, Italy as a primary security system, a rupture disk was installed. All parameters are controlled by a distributed control system (DCS).

The effect of the various operating conditions such as pressure (P) in the range 250–550 bar, CO_2_ flow rates 7.24 and 14.48 g/min, temperature (T) 50 and 65 °C and extraction was carried out for 120 min (six extraction cycles). Each cycle has a static extraction time of 20 min; therefore, using six extraction cycles, the extraction process was completed in 120 min.

The effect of each set of operating conditions was investigated on lutein, lipids, and FAs recovery using following Equation (1):

(1) $$\mathrm{Extraction}\;\mathrm{yield}\;(\mathrm{mg}/g)=W_{C,i}/W_{M},$$

where *W_C,i_* is the extracted weight of lutein, lipids, and FAs (mg) as a function of the extraction time; *W_M_* is the weight of microalgae on a dry basis (g).

The recovery percentage and purity of each intracellular compound were calculated using Equations (2) and (3) [32]:

(2) $$\mathrm{Recovery}\;\left(\%\right)=W_{B}/W_{T}\times 100,$$

(3) $$\mathrm{purity}\;\left(\%\right)=W_{B}/W_{E}\times 100,$$

where *W_B_* is the weight of each extracted intracellular compound (mg) as a function of the extraction time; *W_T_* is the initial weight of each intracellular compound (mg); *W_E_* is the total weight of the extract (mg).

Each experimental condition was investigated three times and for each value the standard deviation (SD) was calculated. After the CO_2_-SF extraction, the extracts were stored in the dark at −80 °C until further analysis.

### 3.5. Analytical Methods

After each CO_2_-SF extraction cycle, the sample was collected in an amber vial and dissolved in 20 mL methanol containing 0.1% butylated hydroxytoluene (BHT) as the antioxidant agent. The methanol-dissolved extract was equally transferred into three different vials for gravimetric analysis, transesterification, and saponification. Then 5 mL of the extracts were gravimetrically quantified, after the complete removal of the solvent using a Zymark TurboVap evaporator (Zymark, Hopkinton, MA, USA). For lutein analysis, saponification was carried out to remove lipids and chlorophylls from the sample, avoiding the overlap of the spectra with the species present in the carotenoid family. In particular, saponification was carried out by adding 1 mL of NaOH solution in methanol (0.05 M) to 5 mL of extract. This solution was left in the dark condition and in an inert atmosphere for 7 h. Once this step was completed, the sample was neutralized with 3 mL of an NH_4_Cl solution in methanol (0.05 M). After saponification, lutein was measured using a u-HPLC Agilent (Santa Clara, CA, USA) 1290 Infinity II with Zorbax reverse phase C18 column with methanol-water (95:5, *v*/*v*) used as a mobile phase solvent, while the sample was dissolved in a methanol/chloroform mixture (90:10) containing 0.1% BHT. The flow rate and column temperature were kept constant at 0.4 mL/min and 28 °C, respectively. For the standard, lutein was purchased from Sigma-Aldrich, St. Louis, MO, USA and used for the quantitative analysis.

The lipid content was extracted from about 120 mg of each lyophilized microalgae using a mixture of chloroform:methanol:distilled water with a ratio of 1:2:0.75 (3.75 mL) in continuous stirring for 1 h using the Bligh and Dyer [40] method, as modified by Tang et al. [41]. After extraction, 1 mL of chloroform and 0.5 mL of double-distilled water were added. The sample was centrifuged at 14,000 rpm for 10 min and the upper phase was taken and stored in a tube. The extraction was repeated on the initial biomass until the color was lost and the extract obtained was combined with the previous extract. The total lipid content was gravimetrically quantified, after the removal of the solvent using a Zymark TurboVap evaporator (Zymark, Hopkinton, MA, USA). For FAs analysis, the extracts obtained after the Bligh and Dyer extraction were transesterified according to the indications given in the standard method UNI ISO 12966-2 [42]. NaOH solution in methanol (0.5 M, 6 mL) and a spatula of boiling chips were added to a known quantity of extract. The sample was transferred to a 50 mL one-mark volumetric Erlenmeyer flask connected to a reflux condenser to boil the sample for about 10 min. At the end of boiling, the apparatus was removed from the heat source and 6 mL of hexane were added from the top of the condenser and then 7 mL of the BF_3_ catalyst in methanol (14%) (B1252, Sigma-Aldrich). The sample was allowed to boil again for 30 min and 5 mL of isooctane were added at the end of the reaction. A 20-mL sample of a saturated NaCl solution were added and swirled, and a second aliquot of saturated NaCl solution was added up to the neck of the flask. The upper layer (2–4 mL) was taken and transferred to a GC glass vial. The chromatographic analysis was carried out using a 7820A GC-FID equipped with an HP-88 100 mt × 0.25 mm × 0.2 µm column. This chromatographic column, produced by Agilent, is composed of a high-polarity bis (Cyanopropyl) siloxane stationary phase and was chosen for its high resolution of positional and geometric isomers of fatty acid methyl esters. According to the chromatographic conditions reported in the standard method UNI ISO 12966-4 [43], the temperature of the injector as well as the detector temperature were maintained at 250 °C. The column was maintained at 120 °C for 5 min and was followed by temperature ramping at 4 °C/min to 240 °C, then held for a further 10 min at 240 °C. Nitrogen (purity ≥ 99.9999%) was used as the carrier gas with a linear velocity of 30 cm/s (flow rate: approximately 1.0 mL/min) and a split ratio of 1:100. The injection volume was 1 µL. The FAs characterization was carried out after extraction in each cycle and an internal analytical standard of the tricosanoic acid (C:23) was used for the quantification of fatty acid methyl esters. To quantify the concentration of the lutein, and FAs compounds, calibration curves were built using chromatographic standards. For standards, a mixture of 37 fatty acid ethyl esters (C4-C24) (Supelco FAME 37, CRM47885, Sigma-Aldrich, St. Louis, MO, USA) was used.

## 4. Conclusions

This study highlighted the potential application of *S. almeriensis* microalga as a natural source of high added-value compounds (lutein and FAs). The experimental outcomes demonstrated that several parameters, including extraction temperature, pressure, and CO_2_ flow rate, played a crucial role in the extraction in terms of recovery and purity. In particular, by increasing the operational pressure and temperature, it was possible to enhance the solubilization of targeting intracellular compounds. However, increasing the CO_2_ flow rate positively influenced both the recovery and purity of lutein and FAs.

The results showed that the maximum cumulative lutein recovery (~98%) with a purity of ~34% was achieved at 65 °C and 55 MPa with a CO_2_ flow rate of 14.48 g/min. In addition, intermediate extraction pressure (40 MPa) supported higher total lipids recovery (18%), while higher pressure (55 MPa) enhanced the FAs recovery (15%) at 65 °C and a CO_2_ flow rate of 14.48 g/min. Furthermore, SF-CO_2_ operating conditions were more favorable for the extraction of C20:0 and C14:1 (ω-5) rather than the other lipids.

Considering the current increase in the demand for SF-CO_2_, this is a suitable method for the extraction of high value-added compounds from microalgae biomass and can be used for the replacement of petrochemicals and harmful solvents still in use for the industrial extraction of the studied compounds. However, the purity of the extracted compounds was lower, so further research needs to be more focused on purity, which may lead to the direct application of high value-added compounds in different human health-related industries.

## Figures and Tables

**Table 1:** Effect of SF-CO_2_ operating conditions on extraction and lipid yield from *S. almeriensis* pretreated biomass.

Operating Conditions			Extraction Yield (mg/g)	Total Lipid Yield (mg/g)
T (°C)	P (MPa)	CO_2_ Flow Rates (g/min)		
50	25	7.24	1.47	1.18
50	40	7.24	6.71	2.05
50	55	7.24	7.37	2.18
65	25	7.24	6.63	1.83
65	40	7.24	15.02	2.72
65	55	7.24	12.42	2.98
50	25	14.48	5.93	1.45
50	40	14.48	9.31	2.79
50	55	14.48	10.13	2.74
65	25	14.48	7.68	2.20
65	40	14.48	8.79	3.71
65	55	14.48	8.74	3.42

Note: Standard deviation was less than 5% in all operating conditions.

**Table 2:** Effect of SF-CO_2_ operating conditions on fatty acids from *S. almeriensis*-pretreated biomass.

Yield of Fatty Acid	IC* (mg/g)	CO_2_ Flow Rate of 7.24 g/min						CO_2_ Flow Rate of 14.48 g/min					
		50 °C			65 °C			50 °C			65 °C		
		Pressure (MPa)											
		25	40	55	25	40	55	25	40	55	25	40	55
C16:0	2.49	0.21	0.31	0.33	0.28	0.38	0.42	0.19	0.39	0.39	0.20	0.48	0.52
C18:0	0.34	0.01	0.03	0.00	0.02	0.04	0.04	0.01	0.04	0.04	0.02	0.04	0.05
C20:0	0.66	0.05	0.15	0.16	0.11	0.23	0.24	0.09	0.24	0.24	0.15	0.33	0.29
C16:1	0.60	0.05	<Ldl	<Ldl	<Ldl	<Ldl	<Ldl	<Ldl	0.01	<Ldl	<Ldl	<Ldl	<Ldl
C17:0	0.41	0.00	0.04	0.04	0.03	0.06	0.06	0.03	0.06	0.08	0.04	0.09	0.08
C18:1	1.09	0.12	<Ldl	<Ldl	0.01	<Ldl	<Ldl	0.01	<Ldl	<Ldl	<Ldl	<Ldl	0.02
C14:1 (ω-5)	0.17	0.01	0.10	0.01	<Ldl	0.16	0.16	0.06	0.16	0.15	0.12	0.02	0.17
C18:2 (ω-6)	1.61	0.04	<Ldl	0.26	0.25	0.01	<Ldl	<Ldl	<Ldl	<Ldl	0.01	0.61	0.01
C18:3 (ω-6)	3.71	0.05	0.09	0.10	0.07	0.14	0.15	0.08	0.18	0.15	0.10	0.01	0.18

Note: IC*: Initial content; <Ldl = lower than the detection limit. Standard deviation was less than 5% at all operating conditions.

**Table 3:** Effect of SF-CO_2_ operating conditions on global extraction yield and recovery of bioactive compounds from pretreated biomass of *S. almeriensis.*

Bioactive Compound	CO_2_ Flow Rate of 7.24 g/min						CO_2_ Flow Rate of 14.48 g/min					
	50 °C			65 °C			50 °C			65 °C		
	Pressure (MPa)											
	25	40	55	25	40	55	25	40	55	25	40	55
LY** (mg/g)	0.13	0.72	0.87	0.55	1.23	1.25	0.10	1.39	2.81	0.43	1.52	2.97
LR*** (%)	6	10	11	9	13	15	7	14	13	11	18	17
FR**** (%)	5	8	8	7	12	12	5	12	12	8	14	15

Note: LY**: Lutein yield; LR***: Lipid recovery; FR****: FAs recovery. Standard deviation was less than 5% in all operating conditions.

**Table 4:** Comparison of the performance of SF-CO_2_ method used in this study with the performance obtained from other methods using green solvent for lutein extraction from several microalgae.

Microalgae	Used Method	Extraction Conditions: T (°C), P (MPa), t (min)	Recovery (%)	Reference
*Scenedesmus almeriensis*	Solid liquid extraction using hexane	na (six stage)	95	[19]
*Scenedesmus* sp.	SF-CO_2_ with 30% of ethanol as co-solvent	70 °C, 40 MPa, and 60 min	76.7	[33]
*Chlorella sorokiniana* MB-1	Solvent extraction using ethanol	35 °C, 0.004 MPa, and 40 min	86.2	[37]
*Chlorella pyrenoidosa*	SF-CO_2_ with 50% of ethanol as co-solvent	50 °C, 25 MPa, and 120 min	87	[38]
*Scenedesmus almeriensis*	SF-CO_2_	65 °C, 55 MPa, and 120 min	98	This study

Recovery of lutein was calculated based on initial content of lutein, which was extracted using standard extraction methods. T: Temperature (°C); P: Pressure (MPa); t: Total extraction time (min); and na: not available.

**Table 5:** Chemical composition of *S. almeriensis.*

Chemical Composition	Concentration (mg/g)
Humidity	89.82
Total dietary fiber (TDF)	225.99
Carbohydrates	45.09
Proteins	129.35
Ash	576.14
Lipids	20.46
FAs	11.17
Lutein	3.04

Note: Standard deviation was less than 5% in all operating conditions.
